# Book Reviews

**Published:** 1902-04

**Authors:** 


### BOOK REVIEWS.

SYPHILIS. A Symposium. E. B. Treat & Co., New York, 1902.

This book of 121 pages is a collection of articles which have appeared in the *International Medical Magazine*, and fairly well covers the phases and relations of the disease. The articles concern the etiology of syphilis, clinical characteristics of the syphilitic chancre, the unrecognized chancre, syphilitic affections of the respiratory tract, of the nervous system, unrecognized syphilis in general practice, syphilis of the stomach, how best to diagnosticate communicative syphilis in a wet nurse, diagnosis and management of syphilis, syphilis of the upper air passages, the curability of syphilis, general remarks on the management of syphilis, treatment of syphilis. These are all live subjects conveniently grouped in book form.

---

MOSQUITO BRIGADES AND HOW TO ORGANIZE THEM. By Donald Ross, F.R.C.S., D.P.H., F.R.S., London. Longmans, Green & Co., New York and London, 1902.

The recognition that the mosquito is the channel of conveyance to human beings of the germs of several of the most important tropical diseases, malaria, yellow fever and elephantiasis, has lead to the necessity for organized effort at exterminating the insects. The function of this book is to point out the best means by which this object may be accomplished. The motto throughout in the work of destruction is the stagnant water, and the methods of practically carrying out the intents of this slogan are described in detail, and at too great length to find place here. The book will serve an eminently practical purpose, and should be read alike by the laity and the medical profession. It sells for the modest sum of 80 cents.

